# Everyday Sadism as a Predictor of Rape Myth Acceptance and Perception of Harassment

**DOI:** 10.1177/0306624X231165430

**Published:** 2023-04-09

**Authors:** Ivonne Alicia Saravia Lalinde, Nicholas Longpré, Melissa de Roos

**Affiliations:** 1 King’s College London, UK; 2 Edge Hill University, Ormskirk, UK; 3 Erasmus University Rotterdam, Netherlands

**Keywords:** sadism, dark tetrad, rape myths, harassment, sexual violence

## Abstract

The #MeToo movement has stressed the need to understand why individuals who witness sexual violence may or may not take action. However, prevention programs usually fail to address the association between personality traits and attitudes, perception, and behavior in the context of sexual violence. To improve prevention programs’ effectiveness, it is vital to understand how personality traits might interfere with willingness to engage in bystander intervention. This study aims to explore the relationships between Everyday Sadism, perception of harassment, Rape Myths and gender in a sample of 177 participants recruited online. Analyses revealed significant gender differences, with men endorsing more Rape Myths, perceiving less harassment, and being more sadistic. Gender and everyday sadism emerged as significant predictors of perception of harassment. In the case of Rape Myths, age emerged as an additional predictor. These results have several implications, ranging from expanding our knowledge of the influence of everyday sadism on factors known to modulate bystander behaviors as well as informing and shaping the development of prevention programs.

## Introduction

Sexual violence includes a wide variety of behaviors, ranging from sexual harassment to sexual coercion, to rape, to sadistic rape, to sexual homicide (Knight et al., 2013). Traditionally, the focus has been on studying the causes of severe forms of sexual violence such as sadistic rape (e.g., Longpré et al., 2020b; Reale et al., 2022) or sexual homicide (e.g., Chopin et al., 2023; Stefanska et al., 2020). The rise of social media movements (e.g., #BeenRapedNeverReported) has shed a light on the high prevalence of less severe forms of sexual violence (de Roos & Jones, 2022a). Recent studies have supported the relationship between subclinical personality traits, sexual violence and underlying offence-supportive cognitions (Beckett & Longpré, 2022). However, few studies have been conducted on less severe forms of sexual violence (e.g., Longpré et al., 2022; Sims-Knight & White, 2018) and our understanding of the nomological network and correlates of these forms of violence is scarce. Therefore, the present manuscript aims to study the relationship between everyday sadism, rape myths acceptance and perception of harassment.

## The Dark Triad and Sadism

Over recent decades, the Dark Triad has contributed to understanding the phenomenon of the human capacity for malevolence (Kowalski et al., 2020) . This constellation of personality traits comprises subclinical narcissism, Machiavellianism, and subclinical psychopathy, which share a common core of social deviance, self-promotion, and emotional coldness (Paulhus, 2014). Narcissism is characterized by an exploitative interpersonal style, a sense of entitlement and superiority, and grandiosity (Jones & Paulhus, 2014). Machiavellianism includes amorality, callousness, manipulativeness, cynicism, and strategic calculating behavior to serve self-interest (Jones & Paulhus, 2014). Finally, psychopathy involves a lack of empathy and remorse, and manipulative, deceitful, and self-centered behaviors (Kreis et al., 2012).

Empirical evidence has supported the addition of everyday sadism as a fourth trait, forming the Dark Tetrad of personality (Paulhus, 2014; Plouffe et al., 2017). Everyday sadism entails aggression that is pleasure-driven and involves the infliction of physical and emotional pain on others to humiliate, punish or control them (Longpré et al., 2020a). In contrast to the important empirical work on the facets of the Dark Triad, research on everyday sadism is more limited, and the concept is often studied from the perspective of criminal behavior and sexual sadism (Buckels et al., 2013; Longpré et al., 2022). However, Longpré et al. (2020b) have shown that sadism extends beyond a sexual disorder found in criminal populations, and in its subclinical form, it can predict maladaptive behaviors and delinquency such as traditional and online bullying (Geel et al., 2017), white-collar crime (Amos et al., 2022), sexual violence (Beckett & Longpré, 2022; Russell et al., 2017), atypical sexual violence (Snow & Longpré, 2022), stalking (Tachmetzidi Papoutsi & Longpré, 2022), and homicide (Monckton-Smith et al., 2017).

Sadism has been identified as a distinct construct despite some similarities and overlapping features with the other facets of the Dark Tetrad (Međedović & Petrović, 2015). Several researchers have argued that these traits, rather than being categorical, are dimensional and present on a spectrum from subclinical to clinical levels (Haslam et al., 2012; Kowalski et al., 2020; Longpré et al., 2020b). Manifestations of cruelty occur to different extents in normal, everyday people (Erickson & Sagarin, 2021), and at similar levels between genders (Longpré et al., 2018). Therefore, the commonality and presence of everyday sadism as a distinct construct have to be accounted for to fully understand the concept of sadism and its impact on cognitions and behaviors (Buckels et al., 2013).

## Harassment

Sexual harassment (SH) is defined as unwelcomed or unwanted conducts, which has the effect or purpose of being hostile, intimidating, humiliating, offensive or degrading (McDonald, 2012). The rationale behind this definition is that not all incidents are necessarily of sexual nature or perpetrated by someone of the opposite sex, but they are usually driven by power and sexism (Leskinen et al., 2011). SH is one of the most prevalent forms of sexual aggression, and extreme forms of harassment have been considered equivalent to rape (Pina et al., 2009). SH is a chronic occupational issue that primarily, but not exclusively, affects women and has led to increasing organizational costs and adverse health and occupational outcomes for victims (Quick & McFadyen, 2017; Shaw et al., 2018). A recent survey found that 22% of those who experienced SH in the workplace had a female perpetrator, while 63% of them were harassed by a male perpetrator (Adams et al., 2020). However, past research into SH has mainly focused on investigating the consequences on the victims, with less thought given to the relationship between harassers’ characteristics and underlying mechanisms (Sims-Knight & White, 2018).

Research on the association between sexual harassment and everyday sadism is scarce, but perpetrators of harassment have been found to be more likely to endorse Rape Myths, to be more coercive, and to be more aggressive than non-harassers (Beckett & Longpré, 2022; Begany & Milburn, 2002; Lucero et al., 2006). These characteristics have, in turn, been linked to everyday sadism (Koscielska et al., 2019; Longpré et al., 2022). Among everyday sadists, greater enjoyment comes from sadistic acts in the absence of consent (Erickson & Sagarin, 2021), which is a component of harassment (McDonald, 2012). Additionally, the link between everyday sadism and online trolling (March & Steele, 2020) also supports a link between everyday sadism and harassment. Online trolling is partly motivated by the reduced pain perception and deficient moral judgments present in everyday sadism (Buckels et al., 2019). The latter predicts harassment proclivity in men (Page & Pina, 2018), and the former could also be a contributing mechanism in harassment. It has also been shown that harassment and sexual violence form part of the same continuum (Knight et al., 2013; Longpré et al., 2020b; Sims-Knight & White, 2018), and sexual violence is, in turn, also associated with sadism (Longpré et al., 2022; Russell et al., 2017). These studies imply that everyday sadism could potentially have a relationship with different forms of harassment and thus highlight the need to investigate this relationship.

## Rape Myths

Sexual assault is a highly prevalent issue. It is estimated that around 20% of women and 4% of men in the United Kingdom (UK) have been sexually assaulted since the age of 16 (Office for National Statistics, 2022), and the police reported the highest number of annual sexual assaults ever recorded at the end of 2021 (63,136 offences; Office for National Statistics, 2022). Despite these alarming data, these figures are likely to be underestimated due to the problem of underreporting of sexually violent crimes (de Roos & Curtis, 2021; de Roos & Jones, 2022a, 2022b), which can include harassment, forced touching, and rape (Astbury & Jewkes, 2010). Many reasons have been found to explain the high number of unreported sexual violence (de Roos & Jones, 2022a, 2022b). One of the factors contributing is Rape Myth acceptance. Rape Myths are false, stereotypical beliefs inclined to trivialize, generalize, or even deny sexual assault (Bonneville & Trottier, 2021) and used to move the blame from the perpetrators to the victims (Bonneville & Trottier, 2021; de Roos & Jones, 2022a, 2022b; Suarez & Gadalla, 2010). It has been argued that Rape Myth endorsement increases the propensity to engage in sexual aggression and the likelihood of engaging in SH among men (Begany & Milburn, 2002; Walfield, 2021).

Positive relationships have also been found between everyday sadism, sexual assault, Rape Myth acceptance, and coercion amongst men (Koscielska et al., 2019; Longpré et al., 2022; Russell & King, 2016). Evidence indicates that highly sadistic individuals might be able to commit these acts and avoid feelings of guilt due to the psychological rationalizations they employ (Longpré et al., 2022). Everyday sadism is thought to originate from defective moral reasoning (Buckels et al., 2019), which could be linked to a greater endorsement of Rape Myths. Boland (2018) found that, out of the Dark Tetrad, sadism had the strongest association with Rape Myth acceptance, particularly acceptance of the “It Wasn’t Really Rape” myth. This relationship’s strength can be attributed to the lack of empathy that sadistic individuals have (Plouffe et al., 2017). These findings suggest that sadism can modulate attitudes toward rape to a greater extent than the other facets of the Dark Tetrad, and influence perception and behavior.

## Bystander Intervention

The *#MeToo* movement and the hashtag *#BeenRapedNeverReported* sought to raise awareness of the prominence of sexual violence and SH, and highlighted its systemic underreporting (Mendes et al., 2019). Victims often remain silent for various reasons, contributing to underreporting (de Roos & Jones, 2022a, 2022b). To overcome this, the focus of several prevention programs has shifted to observers or bystanders (McMahon & Banyard, 2012). The intervention of these third-party witnesses can play a crucial role in reducing sexual violence and SH by stopping events as they occur, reporting observed events, and delivering negative feedback to perpetrators (McMahon & Banyard, 2012). In contrast, SH in the workplace can be encouraged by bystanders’ non-intervention (Bowes-Sperry & O’Leary-Kelly, 2005), and data gathered from university students has revealed that a third reported witnessing sexual violence on campus, but only 53% of them chose to intervene (Revolt Sexual Assault, 2018).

Bystander intervention can be influenced by factors such as gender and personality traits. For example, women are more likely to intervene (de Roos & Jones, 2022a), and dark traits are associated with barriers to intervention (Lyons et al., 2022). Whilst a link between everyday sadism and bystander intervention is yet to be established, several characteristics of this trait could impair such intervention. The higher levels of Rape Myth endorsement and the lack of empathy, agreeableness, and prosocial behaviors are features of sadism that can support bystander passivity (Kowalski et al., 2020; Leone et al., 2021; Međedović & Petrović, 2015).

## Aims

Recent social media movements have highlighted the high prevalence of lower forms of sexual violence (de Roos & Jones, 2022a; Mendes et al., 2019), but also emphasized the lack of research on the correlates (Sims-Knight & White, 2018), limiting our ability to design effective prevention programs. As a result, an emerging area of prevention is now focusing on bystander intervention (McMahon & Banyard, 2012), which attempts to make sexual violence a matter of collective responsibility (Lipnic, 2016). Bystander intervention training seeks to provide individuals with knowledge about sexual violence and the necessary skills to confidently intervene and interrupt witnessed problematic events, as well as reducing the likelihood of committing sexual violence (Banyard & Moynihan, 2011). However, these prevention programs fail to acknowledge the association between personality traits and attitudes, perception, and behavior in the context of sexual violence (Beckett & Longpré, 2022). To improve our effectiveness in preventing sexual violence, it is vital to understand which individuals’ characteristics increase the risk of committing sexual violence or represent a barrier to bystander intervention. Consequently, it is important that research aims at bridging the gaps in our knowledge and explores the correlates of lower forms of sexual violence and its perception.

Therefore, the present study aims to explore the relationships between everyday sadism, perception of harassment, and Rape Myth acceptance. Furthermore, the impact of gender will be studied. Based on the previous literature, it is hypothesized that individuals that have higher sadistic traits will be less likely to perceive harassment in different situations and will endorse more Rape Myths than those at the lower end of the sadistic spectrum. Further, it is hypothesized that men will perceive less harassment, endorse more Rape Myths, and present higher levels of everyday sadism when compared to women.

## Methods

### Participants

The original sample was composed of *N* = 201 participants from England & Wales. Following a clean-up, as some respondents did not complete the full survey, the final sample was reduced to *N* = 177, who were recruited on social media (*n* = 132) and Sona (*n* = 45). The mean age of participants was 28.86 years old (*SD* = 12.22; Range = 18–85). The majority of participants were women (*n* = 105; 59.3%), white (*n* = 116; 65.5%), heterosexual (*n* = 147; 83.1%) and single (*n* = 92, 52%). See Table 1 for full demographic data.

**Table 1.** Sample Demographics.

	*n*	%
Age: mean = 28.86 years; *SD* = 12.22; range, 18–85		
Gender		
Male	71	40.1
Female	105	59.3
Prefer not to say	1	.6
Total	177	100
Sexual orientation		
Heterosexual	147	83.1
Homosexual	9	5.1
Bisexual	15	8.5
Other	1	.6
Prefer not to say	5	2.8
Total	177	100
Relationship status		
Long-term relationship	54	30.5
Short-term relationship	31	17.5
Single	92	52.0
Total	177	100
English fluency		
Fluent	157	88.7
Not fluent	20	11.3
Total	177	100
Ethnicity		
White	116	65.5
Mixed/multiple ethnicities	15	9
Asian	9	5.1
Indian	2	1.1
Black/African/Caribbean	6	3.4
Other ethnic groups	29	16.4
Total	177	100

### Ethics and Procedure

The project has received ethical approval by a university in England & Wales. Consent form, socio-demographic questions, scales, and debrief form were added in Qualtrics, a web-based tool that allows the creation and distribution of surveys. Qualtrics allowed for complete anonymity, as respondents and researchers are never in contact. Part of the participants were recruited in one university through SONA system, a cloud-based participant management software that allows researchers to set up online studies, recruit participants, and manage credits or paid participation. Their participation did not involve any financial compensation, but they were awarded 1.5 credits for completing the survey. A second part of the participants were recruited on professional social media platforms (i.e., Twitter & LinkedIn) and personal social media platforms (i.e., Facebook).

Participants were presented with a detailed consent form that gave an overview of the study, as well as the necessary information. A clear warning about the sensitive nature of the study, in red and bold font, was presented in the consent form. Participants were also informed of their rights and the anonymous nature of the survey. At the end of the survey, participants were provided with resources for victims of sexual violence, both on- and off-campus supports.

### Scales

#### Positive Image Scale (PIS)

This nine-item scale measures the participants’ likelihood of portraying themselves in an overly positive manner to others (MIDSA, 2011). Items are rated on a 5-point Likert-style scale, ranging from “1—definitely false” to “5—definitely true,” with higher scores indicating a higher probability of responding in a socially desirable manner. An example of an item is “I always obey laws even if I'm unlikely to get caught.” In this study, the Cronbach’s alpha was .65.

#### Lack of Perspective Taking (LPT)

This eight-item scale measures perspective-taking ability and the propensity to do so (MIDSA, 2011). Lack of perspective-taking has been associated with victim-blaming and Rape Myths (Gravelin et al., 2019). Items are rated on a 5-point Likert-style scale, ranging from “1—definitely false” to “5—definitely true,” with higher scores indicating a lower probability of considering others’ perspective. An example of an item is “I find it difficult to see things from the “other guy’s” point of view.” In this study, the Cronbach’s alpha was .41.

#### Short Sadistic Impulse Scale (SSIS)

This 10-item scale measures sadistic inclination and the propensity to be cruel toward other individuals at a sub-clinical level (O’Meara et al., 2011). Items are rated on a 4-point Likert-style scale, ranging from “1—strongly agree” to “4—strongly disagree”, with lower scores indicating higher sadistic tendencies. An example of an item is “I have humiliated others to keep them in line.” The Cronbach's alpha was .83.

#### Updated Illinois Rape Myth Acceptance Scale (uIRMA)

This 22-item scale (McMahon & Farmer, 2011) is an updated version of the IRMA by Payne et al. (1999). It measures acceptance of victim-blaming attributions and rape-supportive cognitions. It contains four subscales: (1) She asked for it (6 items), (2) He didn’t mean to (6 items), (3) It wasn’t really rape (5 items), and (4) She lied (5 items). Items are rated on a 4-point Likert-style scale, ranging from “1—strongly agree” to “4—strongly disagree,” with lower scores indicating higher endorsement of Rape Myths. An example of an item is “If a girl goes to a room alone with a guy at a party, it is her own fault if she is raped.” In this study, the Cronbach’s alpha was .93.

#### Perception Toward Harassment (PTH)

This 24-item scale was developed from the Harassment Scale (MIDSA, 2011). This amended version measures perception of harassment rather than harassment behaviors (Longpré et al., 2022). Items are rated on a 4-point Likert-style scale, ranging from “1- strongly agree” to “4- strongly disagree”, with higher scores indicating better perception of harassment. An example of an item is “I think it’s acceptable to spread rumors about others.” In this study, the Cronbach’s alpha was .93.

### Analyses

Power analyses, using G*Power software, were conducted to assess the sample size, and revealed that it was sufficient to uncover large to medium effect size across analyses. Skewness and kurtosis values ranged between −2 and +2, which supported the normal distribution assumption (George & Mallery, 2010); therefore, parametric analyses were conducted.

All statistical analyses were performed using the Statistical Package for Social Sciences (SPSS) v26 (IBM, New York, USA). Independent *t*-tests were carried out to assess gender differences on everyday sadism, perception toward harassment, and Rape Myth acceptance. Then, Pearson correlations were conducted to investigate the relationships between scale total scores. Finally, two multiple linear regressions were performed to predict the explanatory variables’ value on perception toward harassment and Rape Myth acceptance.

## Results

### Independent Samples *t*-Test

Independent samples *t*-tests were conducted to assess gender differences across the main scales of interest. For all scales, statistically significant gender differences were found. The full results can be found in Table 2.

**Table 2.** Results of Independent Sample *t*-Tests and Descriptive Statistics for Main Scales by Gender.

Outcome	Group						*t*	*df*
	Females			Males				
	*n*	*M*	*SD*	*n*	*M*	*SD*		
SSIS	103	36.49	4.13	67	34.55	4.45	−2.89**	168
PTH	93	83.75	5.96	61	78.23	10.07	−3.86***	87.70
uIRMA	97	78.57	8.64	69	69.20	10.28	−6.35***	164
Subscale 1 (uIRMA)	103	21.49	2.95	71	18.96	3.56	−4.93***	131.72
Subscale 2 (uIRMA)	103	20.96	3.04	69	18.83	3.10	−4.48***	170
Subscale 3 (uIRMA)	103	19.16	1.89	71	17.42	2.70	−4.67***	116
Subscale 4 (uIRMA)	103	16.92	3.03	71	13.94	3.27	−6.17***	172

** *p* < .01. ***p < .001.

Independent samples t-tests on the SSIS indicated that men (*M* = 34.55; *SD* = 4.45) have higher sadistic tendencies than women (*M* = 36.49; *SD* = 4.13); *t*(168) = -2.89, *p* = .004. Independent samples *t*-tests on the PTH scale indicated that women (*M* = 83.75; *SD* = 5.96) have a greater perception of harassment than men (*M* = 78.23; *SD* = 10.07); *t*(87.70) = −3.86, *p* < .001.

Independent samples *t*-tests on the uIRMA scale indicated that men (*M* = 69.20; *SD* = 10.28) endorse more Rape Myths than women (*M* = 78.57; *SD* = 8.64); *t*(164) = −6.35, *p* < .001. A breakdown of the uIRMA revealed that this gender difference was found in every subscale. Subscale 1: males (*M* = 18.96; *SD* = 3.56) and females (*M* = 21.49; *SD* = 2.95); *t*(131.72) = −4.93, *p* < .001. Subscale 2: males (*M* = 18.83; *SD* = 3.10) and females (*M* = 20.96; *SD* = 3.04); *t*(170) = −4.48, *p* < .001. Subscale 3: males (*M* = 17.42; *SD* = 2.70) and females (*M* = 19.16; *SD* = 1.89); *t*(116) = −4.67, *p* < .001. Subscale 4: males (*M* = 13.94; *SD* = 3.27) and females (*M* = 16.92; *SD* = 3.03); *t*(172) = -6.17, *p* < .001.

### Pearson-Correlations

Pearson correlations were conducted to measure the relationships between the scales. The results are presented in Table 3. The SSIS was positively and significantly correlated with the PIS (*r* = .26, *p* < .001), PTH (*r* = .47, *p* < .001), and with the uIRMA (*r* = .41, *p* < .001) and its subcomponents (correlations ranging from .17 to .41). PTH had a significant, negative correlation with LPT (*r* = −.21, *p* = .010) and significant, positive correlations with the PIS (r = .16, *p* = .046), the uIRMA (*r* = .51, *p* < .001), and its subcomponents (correlations ranging from .37 to .51). Lastly, LPT was negatively associated with the PIS (*r* = −.27, *p* < .001).

**Table 3.** Correlations Between All Scales.

Variables	1	2	3	4	5	6	7	8	9
1. SSIS	—	.26**	−.15	.47***	.41***	.41***	.17*	.34***	.39***
2. PIS		—	−.27***	.16*	.04	.02	.02	.01	.06
3. LPT			—	−.21*	−.02	−.02	−.03	.01	−.03
4. PTH				—	.51***	.47***	.37***	.51***	.41***
5. uIRMA					—	.87***	.78***	.81***	.86***
6. Subscale 1 (uIRMA)						—	.54***	.68***	.64***
7. Subscale 2 (uIRMA)							—	.50***	.54***
8. Subscale 3 (uIRMA)								—	.62***
9. Subscale 4 (uIRMA)									—

* *p* < .05. **p < .01. ***p < .001.

### Multiple Linear Regression

Finally, two multiple linear regressions were conducted to analyse the predicting values of gender, age, PIS, LPT, and SSIS on PTH (see Table 4) and the uIRMA (see Table 5). The first regression model was statistically significant and explained 24.4% of the variance in PTH, R^2^ = .24, *F*(5, 139) = 8.97, *p* < .001. Gender, β = −.23, *t*(139) = -3.08, *p* = .003, and the SSIS, β = .36, *t*(139) = 4.66, *p* < .001, were both significant predictors of PTH. All remaining variables were not significant predictors of PTH.

**Table 4.** Multiple Linear Regression Analysis for Variables Predicting Perception Toward Harassment.

Model	Unstandardized coefficients		Standardized coefficients	*t*	Significance	Collinearity statistics	
	*B*	*SE*	Beta			Tolerance	Variance inflation factor (VIF)
Age	0.00	0.05	.00	0.03	.976	0.97	1.04
Gender (men)	−3.61	1.17	−.23	−3.08	.003	0.95	1.06
SSIS	0.62	0.13	.36	4.66	.000	0.90	1.19
LPT	−0.20	0.16	−.10	−1.26	.210	0.93	1.08
PIS	0.00	0.10	.00	0.03	.973	0.87	1.15

*Note.* Dependent variable: Perception Toward Harassment (PTH; *r* = .49, *r*^2^ = .24, Adj *r*^2^ = .22).

**Table 5.** Multiple Linear Regression Analysis for Variables Predicting Rape Myth Acceptance.

Model	Unstandardized coefficients		Standardized coefficients	*t*	Significance	Collinearity statistics	
	*B*	*SE*	β			Tolerance	Variance inflation factor (VIF)
Age	−0.19	0.06	−.23	−3.46	.001	0.95	1.08
Gender (men)	−7.23	1.35	−.37	−5.37	.000	0.93	1.08
SSIS	0.74	0.16	.33	4.66	.000	0.88	1.13
LPT	.05	0.19	.02	0.25	.802	0.93	1.07
PIS	−0.01	0.12	−.00	−0.05	.962	0.86	1.17

*Note.* Dependent variable: Rape Myth acceptance (uIRMA; *r* = .61, *r*^2^ = .37, Adj *r*^2^ = .35).

The second regression model was statistically significant and explained 37.3% of the variance in the uIRMA, R^2^ = .37, *F*(5, 145) = 17.27, *p* < .001. Age, β = −.23, *t*(145) = −3.46, *p* = .001, gender, β = −.37, *t*(145) = −5.37, *p* < .001, and the SSIS, β = .33, *t*(145) = 4.66, *p* < .001, were all significant predictors of the uIRMA. All remaining variables were not predictors.

## Discussion

### Overview of the Results

This study aimed to explore the relationships between everyday sadism, perception toward harassment, and Rape Myth acceptance. The effect of external correlates and gender was also measured. Analyses revealed gender differences between men and women in everyday sadism, perception of harassment, endorsement of Rape Myths, and in all the subscales of Rape Myth acceptance. These findings suggest that men endorse more Rape Myths, are worse at perceiving harassment, and show more sadistic tendencies than women. These results partially support the hypotheses, as gender differences were not expected for everyday sadism.

Correlational analyses revealed associations between everyday sadism, perception of harassment, and Rape Myth acceptance. These results indicate that highly sadistic individuals have a worse perception of harassment and are more likely to endorse Rape Myths than those at the lower end of the sadistic spectrum; these findings support the hypothesis. A relationship was found between Rape Myth acceptance and perception toward harassment, which links these forms of sexual violence and suggests that those who endorse fewer Rape Myths have a better perception of harassment.

The first regression model revealed that being highly sadistic and male could predict a lower perception of harassment. The second regression model indicated that being highly sadistic, male, and older could predict the acceptance of more Rape Myths. These findings are consistent with the hypotheses.

### Implications

#### Everyday Sadism and its Correlates

The main objective of this study was to explore the relationships between everyday sadism, perception of harassment, Rape Myths, and external correlates. Although most findings are consistent with previous research, there are some that conflict with past literature. The association between everyday sadism and perception of harassment had not been previously explored, but due to their link to sexual violence (e.g., Russell et al., 2017; Sims-Knight & White, 2018), a relationship between these variables was expected. Perpetrators of harassment have characteristics such as coercion, aggression, and the acceptance of Rape Myths (Begany & Milburn, 2002; Lucero et al., 2006), which are also related to everyday sadism (Koscielska et al., 2019; Longpré et al., 2022; Reidy et al., 2011; Russell & King, 2016). This can explain highly sadistic individuals’ poor perception of harassment. If observers of harassment identify personal similarities with perpetrators, they will display more empathy toward perpetrators rather than victims (de Roos & Jones, 2022a). Similarly, the relationship between being less sadistic and responding in a socially desirable manner is consistent with this trait. As sadistic tendencies decrease, individuals might be more interested in being viewed favorably by others and behave in a more prosocial fashion (Plouffe et al., 2019).

The callousness, lack of empathy, and cruelty characteristic of sadism were evidenced in this study by the strength of its association with the “She asked for it” subscale. This result supports the original hypothesis from Boland (2018), which stated that everyday sadism would have the strongest correlation with the “She asked for it” subscale. However, the findings reported by the author indicated a stronger association with the “It Wasn’t Really Rape” myth. Differences in the results might be explained by the participants that were included in this study. Boland (2018) recruited predominantly female university students from the United States in their early 20s, whilst the current sample was more diverse, with most participants being recruited outside of UK universities, with a larger male representation and an older average age. This is also consistent with the effects that older age and being male had on Rape Myths.

Regarding the perception of harassment, its link with lack of perspective-taking suggests that the ability to perceive a situation from others’ points of view is involved in the capacity to perceive harassment. In contrast, the absence of this ability has been associated with victim-blaming (Gravelin et al., 2019), and can hinder the likelihood of viewing a harassment situation as problematic (Leone et al., 2021). Therefore, previous research supports this association.

The association between the perception of harassment and the Positive Image Scale can be attributed to the fact that those who answer in a socially desirable manner might be more inclined to project favorable images of themselves (MIDSA, 2011). For this reason, they may also identify harassment situations as socially undesirable and problematic. Further support for this notion comes from the link that was found between the Positive Image and the Lack of Perspective Taking Scales. Higher social desirability was associated with more perspective taking, a skill that would be necessary when making a judgment about the social acceptability of a situation, in this case, of harassment.

While harassment and rape are considered as different types of sexual violence, recent studies have supported the notion that they are part of the same continuum of sexual violence (Knight et al., 2013; Longpré et al., 2020b). In other words, they are not different in kind (Taxon; for more details, see Longpré et al., 2023), but are different levels of severity and involvement into sexual violence. This is consistent with the correlation that was found between Rape Myths and perception of harassment. This result also supports the idea that these subdivisions of sexual violence are components of an Agonistic Continuum (Knight et al., 2013; Longpré et al., 2020b).

#### Impact on Bystander Intervention

All together, results illustrate how personality traits, specifically everyday sadism, can modulate attitudes and perception, and partially explain why they might be a barrier to bystander intervention. They can also be used to help understand why some individuals choose to remain passive whilst others intervene. Although prevention programs tend to be directed to groups and do not focus on specific individuals’ characteristics, the results produced in this study can inform these programs about the elements that could hinder bystander intervention and help improve their effectiveness. For instance, the lack of empathy, impaired emotion recognition (Pajevic et al., 2018), and lack of prosocial behaviors (Međedović & Petrović, 2015) that are characteristic of sadism could be focused on. However, it has to be noted that to change attitudes, with the ultimate goal of changing behaviors, requires profound, long-term commitment (Heisecke, 2014). Therefore, addressing these elements might affect those at the lower end of the sadistic spectrum and help increase their bystander intervention. However, deeper measures would be necessary to successfully encourage bystander intervention for those at the upper end of the sadistic spectrum. Lastly, this study has contributed to understanding the relationship between everyday sadism and harassment, which has not been widely explored in past literature.

### Gender

Another objective of this study was to measure the effect of gender. Research into SH and sexual assault suggests that perpetrators of these crimes target all genders, but women are the primary victims (Macik-Frey et al., 2007). In contrast, perpetrators tend to be male, and this trend becomes more evident as the severity of sexually violent crimes increases (Longpré et al., 2018). The higher endorsement of Rape Myths and worse perception of harassment observed in men support past findings from Fakunmoju and Bammeke (2017) and Cortina and Berdahl (2008) respectively. Gender was one of the best predictors of harassment and, for Rape Myths, it was even a slightly better predictor than everyday sadism. The gender differences that were found for everyday sadism, although small, reached significance and contradict previous findings by Buckels et al. (2013) and Longpré et al. (2018). This result calls for further expansion of the literature on everyday sadism and gender differences.

A possible explanation for the gender differences can be found in Davies and Rogers (2009), who reported that male bystanders assign more blame to female victims than female bystanders because they do not relate to female victims as much as do the female. In this study, all the Rape Myth items contained examples of female victims only, which might have led female participants to identify more with victims and endorse fewer myths. Regarding the perception of harassment, male participants might have identified more similarities with the perpetrators as they are usually male (de Roos & Jones, 2022a, 2022b), and for this reason, they were less able to perceive harassment in various situations.

Another alternative explanation for the gender differences in this study is that women tend to have closer encounters with traumatic experiences than men, either through the experiences of others close to them or their own experiences (de Roos & Jones, 2022a). Therefore, women might have more knowledge of sexual violence, leading them to endorse fewer Rape Myths and perceive more situations as harassment. This could explain why women are more likely to intervene than men (de Roos & Jones, 2022a, 2022b). In fact, greater knowledge of sexual violence has been found to significantly predict bystander intervention (Banyard, 2008; McMahon, 2010). Prevention programs already educate on sexual violence (Banyard & Moynihan, 2011). However, due to these significant gender differences, it could be beneficial to have more in-depth training for men, emphasizing perspective taking.

#### Age

This investigation measured the direct impact of everyday sadism on Rape Myth acceptance and perception of harassment when other control variables that could influence their prediction were included. The absence of an age effect on the perception of harassment concurs with previous studies (e.g., Foulis & McCabe, 1997). Similar to McCabe and Hardman (2005), age in the present study was not a significant predictor of perception of harassment. This suggests that age might not be as influential as other individual factors in the perception of harassment. However, it has to be noted that other studies have found differences in perceptions of harassment between age groups. Reese and Lindenberg (2005) found that older male and female employees are more knowledgeable about organizational sexual harassment policies and procedures than younger ones. These authors, however, had a sample that was on average considerably older than the current sample and those included in the studies that produced non-significant results. Therefore, older age groups might not have had sufficient representation to impact the present results. Overall, there is limited research into this association, and this reflects a need for future investigations with more diverse samples.

By contrast, age emerged as a significant predictor of Rape Myths: the second model revealed that older participants endorsed more Rape Myths than younger ones. This is consistent with prior findings (e.g., Walfield, 2021), and it can also be linked to knowledge of sexual violence. In recent years, online movements such as the *#MeToo* sought to raise awareness of and knowledge about sexual violence and SH (Mendes et al., 2019). However, as the evidence suggests that younger individuals use social media platforms significantly more than older individuals (Ortiz-Ospina, 2019), younger people are more likely to have been exposed to these initiatives, increasing their knowledge of sexual violence and its widespread nature.

Behaviors that are now considered unequivocally unacceptable used to be tolerated and even justified (Cortina & Berdahl, 2008). It could be that older participants have been more isolated from recent social movements and have not had as much exposure to more enlightened ways of viewing sexual violence. This highlights the importance of sexual education in prevention programs. However, education alone might not be sufficient to promote intervention behaviors in older adults, as the literature shows that they are less likely to initiate behavioral changes than younger adults (Carstensen & Hartel, 2006). Carstensen and Hartel (2006) argue that to successfully motivate change in this population, it is necessary to communicate persuasive messages tailored to older generations, so they can perceive the information as self-relevant and appealing. Overall, this result should inform prevention programs about the role of age in bystander intervention.

### Limitations

The first limitation of this study is the low reliability of two scales. Cronbach’s alpha values of .70 or above indicate acceptable reliability (Abraham & Barker, 2015). However, the values obtained for the Positive Image and the Lack of Perspective Taking scales were below the recommended ones, especially in the latter. It was decided that they would be used in the final analyses as they were only control scales, but this might have led to some unexpected results. For example, lack of perspective taking has been associated with both Rape Myths (Gravelin et al., 2019), and everyday sadism (Pajevic et al., 2018), but such associations were not found in this study, and this could be a product of the low reliability of the scale.

A second limitation of this study stems from the large proportion of participants who were university students, with a majority of them from the same university and area in England & Wales. This could affect the extension of the results and conclusions to the general population, as students from higher education institutions only represent around 3.6% of the UK population (Office for National Statistics, 2022). However, it is pertinent to research harassment and Rape Myths in a university sample, as nearly two in every three UK university students and graduates report having experienced a form of sexual violence (Revolt Sexual Assault, 2018). It is worth noting that most of the results are consistent with those reported in previous research, so the effect of this limitation might have been minimal.

## Conclusion

This study found that everyday sadism, Rape Myth acceptance, perception of harassment, and gender were associated. Men displayed more sadistic tendencies, perceived less harassment, and endorsed more Rape Myths than women. Everyday sadism had a relationship with perception of harassment and Rape Myth acceptance. Higher sadistic tendencies and being male were predictors of a lower perception of harassment, and for Rape Myth acceptance, older age emerged as an additional predictor of more Rape Myth endorsement.

Bystander intervention is an emerging area of prevention programs that can play a key role in reducing forms of sexual violence, such as harassment, by making these crimes a matter of collective responsibility. Although research in the field has identified several barriers to intervention, it has not yet explored the influence of socially aversive personalities on factors that are known to modulate bystander behaviors. This study can be used to inform and shape the development of prevention programs. Further, it provides support for past literature and expands on the limited knowledge about the link between everyday sadism and harassment. Future research should attempt to identify other variables that can hinder bystander intervention and build on the results yielded in this study. Only when addressing these precursors, can we effectively combat sexual violence as a society.
